# Molecular and cellular insights into Zika virus-related neuropathies

**DOI:** 10.1007/s13365-017-0514-3

**Published:** 2017-01-26

**Authors:** Kai Zhou, Long Wang, Di Yu, Hesuyuan Huang, Hong Ji, Xuming Mo

**Affiliations:** 1 Department of Cardiothoracic Surgery, Children’s Hospital of Nanjing Medical University, Nanjing, 21008 China; 2 Department of Infectious Disease, Children’s Hospital of Nanjing Medical University, Nanjing, 210008 China; 3 Jiangsu Provincial Center for Disease Control and Prevention, Nanjing, 210008 China

**Keywords:** Zika virus, Guillain–Barré syndrome, Microcephaly, Neurodevelopment

## Abstract

Zika virus (ZIKV), a relatively elusive *Aedes* mosquito-transmitted *flavivirus*, had been brought into spotlight until recent widespread outbreaks accompanied by unexpectedly severe clinical neuropathies, including fetal microcephaly and Guillain–Barré syndrome (GBS) in the adult. In this review, we focus on the underlying cellular and molecular mechanisms by which vertically transmitted microorganisms reach the fetus and trigger neuropathies.

## Introduction

The tropical pathogen Zika virus (ZIKV), an *Aedes* mosquito-borne *flavivirus*, is an emerging arbovirus, initially discovered in Uganda as early as 1940s, but the geographic range has expanded drastically, marching across Latin America, Africa, and Southeast Asia, between January 1, 2007 and March 1, 2016 (Haddow et al. 2012; Wikan and Smith 2016). ZIKV remained relatively elusive due to the most asymptomatic and quiet moderate nature and of the infection, and, when self-limited febrile symptoms are present, typically characterized by skin rash, conjunctivitis, and joint pain, just resembling that of dengue fever and chikungunya (Brasil and Nielsen-Saines 2016). However, recent widespread outbreaks had brought ZIKV into spotlight, as evidence denoting that ZIKV has been causally associated with adverse fetal microcephaly in newborns (Malkki 2016a) and severe neurological injuries in adults including Guillain–Barré syndrome (Malkki 2016b; Parra et al. 2016; van den Berg et al. 2014). In addition to the increase of severe illnesses, novel modes of ZIKV deliveries have been detected, including sexual transmission (Hills et al. 2016) and maternal-fetal transmission (Brasil and Nielsen-Saines 2016; Brasil et al. 2016b). Presence of ZIKV in the male semen and female genital tract poses notable challenges, implying that ZIKV can be spread through sexual intercourse and by vertical ransmission (D’Ortenzio et al. 2016; Mansuy et al. 2016; Petersen et al. 2016; Prisant et al. 2016). ZIKV was reported in the amniotic fluid and placenta of pregnant women whose fetuses had been diagnosed with microcephaly, and microcephalic fetal neural tissues, indicating that ZIKV can breach the placental barrier and invade the fetus (Calvet et al. 2016; Martines et al. 2016; Mlakar et al. 2016).

The explosive and unprecedented cases of microcephaly and Guillain–Barré syndrome prompted the World Health Organization (WHO) to declare a Public Health Emergency of International Concern on February 1, 2016 (Heymann et al. 2016) and to advocate researches into possible causal relationships and underlying mechanisms of ZIKV-induced neurologic disorders.

## Molecular insights into ZIKV

Despite epidemic in Africa and Asia during the past half century, ZIKV infections were not linked to severe human disorders until now. It has been postulated that genetic variations of the ZIKV genome may have contributed to its accelerated transmissibility and infectivity, enhanced neurotropism, and increased replicative capacity. Due to the remaining elusive reasons for this, molecular insight into this virus may benefit our understanding of the biology of ZIKV and vaccines development.

ZIKV is an enveloped flavivirus with a nearly 10,800 nucleotides positive-sense RNA molecule. The derived single polyprotein is processed after translation into three structural proteins (capsid [C], precursor Membrane [prM], and envelope [E]) and 7 nonstructural proteins (NS1, NS2A, NS2B, NS3, NS4A, NS4B, and NS5) by host and viral proteases (Kuno and Chang 2007). Structural protein C interacts with the viral RNA to construct a nucleocapsid, prM inhibits premature viral fusion with host membranes, and E, the viral fusogen, regulates cellular attachment, accelerates membrane fusion, and releases the viral genome into the host (Lazear and Diamond 2016; Mukhopadhyay et al. 2005), whereas the viral nonstructural proteins mediate viral transcription and replication and also mitigate host antiviral responses (Diamond and Pierson 2015; Lazear and Diamond 2016; Mukhopadhyay et al. 2005; Suthar et al. 2013).

To inspect the molecular evolution of ZIKV, Wang L et al. (2016) conducted phylogenetic and genetic studies. Interestingly, two major lineages of ZIKV, African and Asian, was detected, whereas the contemporary human strains derive from the Asian lineage (Faria et al. 2016; Wang et al. 2016). Numerous genetic variations in ZIKV genomes has been revealed, along of which ZIKV has evolved from causing only a benign disease to trigger adverse neurological disorders (Baronti et al. 2014; Wang et al. 2016). Structural modeling studies suggest that genetic variations of ZIKV could elicit specific structural changes of prM protein, which could play a role in neurotropism and neurovirulence. Future experiments are eagerly required to determine how such amino acid or nucleotide substitutions impact the increased epidemic potential, neurotropism, and pathogenesis.

ZIKV is closely linked to dengue virus, belonging to the family *Flaviviridae*. Both genetically and serologically, ZIKV is associated with the four serotypes of dengue with approximately 43% amino acid sequence consensus (Lanciotti et al. 2008). Intriguingly, several antibodies isolated from dengue patients potently neutralize ZIKV through targeting a conformational epitope (Barba-Spaeth et al. 2016; Pierson and Graham 2016). The structural basis of potent Zika–dengue virus antibody cross-neutralization was revealed by comparing the Zika and dengue virus immunocomplexes, providing a lead for rational, epitope-directed design of a universal vaccine enough for inducing potent cross-reactivity antibodies to opposing Zika and dengue viruses simultaneously (Barba-Spaeth et al. 2016).

## Animal models developed for ZIKV researches

In light of the catastrophic outcomes of this rapidly emerging infectious disease, it is urgently required to develop animal models of in utero ZIKV infection, which facilitate to define the plausibility of causal relationships between ZIKV and neurological diseases, and to assess the mechanisms of congenital abnormalities (Broutet et al. 2016; Rasmussen et al. 2016). In humans, the placenta plays a pivotal role in preventing vertical transmission of viruses (Coyne and Lazear 2016; Pastula et al. 2016). The question of whether the placenta is permissive to viral breaching has been challenging to resolve and confirm the mode of sexual and maternal-fetal transmission of ZIKV.

Several particularly novel and relevant mouse models, the most powerful tools for biomedical researches by far, are established to unravel the mysteries of placental breaching and transmission. ZIKV invades multiple primary human placental cell types and explants, suggesting placental and paraplacental routes of virus mother-to-fetus transmission routes (Quicke et al. 2016; Tabata et al. 2016). Lazear et al. (2016) established a mouse model lacking interferon α/β receptor 1 (*Ifnar1* ^−/−^), which succumb to infection and exhibits neurological destruction, accompanied by high viral loads in the brain and spinal cord, as well as in the spleen, testes, and serum, which is consistent with severe symptoms of ZIKV in humans, as did a IFN regulatory factor (*Irf*)*3* ^−/−^ *Irf5* ^−/−^ *Irf7* ^−/−^ triple knockout mouse strain that are deficient for type I IFN production (Lazear et al. 2016). Genetic deletion and antibody blockade of Ifnar1 early in pregnancy causes infection of the placenta and embryonic brain, leading to in utero growth restriction and spontaneous pregnancy losses reported in ZIKV-infected pregnant women (Miner et al. 2016a; Suter and Aagaard 2016). Li et al. (2016b) utilizes *Irf3* ^−/−^ *Irf5* ^−/−^ *Irf7* ^−/−^ triple knockout strain to inoculate virus into the peripheral circulation by retro-orbital injection, rather than direct injection into the brain, to simulate the blood-borne transmission (Li et al. 2016b).

Moreover, a nonhuman primate model was established to closely emulate human pathologies by injecting a current ZIKV strain subcutaneously in five locations on the forearms of a pregnant pigtail macaque at 119 days gestation, which is beneficial for the development of therapeutics and vaccines that could attenuate intrauterine infection of ZIKV (Abbink et al. 2016; Adams Waldorf et al. 2016; Dowd et al. 2016). Periventricular lesions were significantly detected in the nonhuman primate model, along with cerebral white matter hypoplasia, periventricular white matter gliosis, and axonal and ependymal injury (Adams Waldorf et al. 2016).

## Cellular and molecular mechanisms implied in ZIKV-induced microcephaly

By far, the global alarm of the Zika virus is its great threat to fetal brain. ZIKV infection and microcephaly are causally associated based on epidemiological data indicating an increased risk of microcephaly coinciding with the ZIKV outbreak (Brasil et al. 2016a; Brasil et al. 2016b; de Fatima Vasco Aragao et al. 2016; Hayes 2009; Sarno et al. 2016). However, definitive proofs are still needed to confirm the causality between the viral epidemic and malformations in fetal brains.

During telencephalic development, neural progenitor cells (NPCs) proliferate, differentiate into numerous fates, migrate to their apposite positions, and maturate into a precisely orchestrated human brain, capable of higher-order language, cognition, and emotion (Budday et al. 2015). Disturbance of these processes resulted in neurodevelopmental disorders including fetal microcephaly, thought to derive from the depletion of NPC population, either through premature differentiation or loss of NPCs (Barkovich et al. 2012; Kriegstein and Alvarez-Buylla 2009; Molyneaux et al. 2007; Wilsch-Brauninger et al. 2016; Wollnik 2010; Woodworth et al. 2012). The NPCs in the fetal brain appear to be probably impacted, due to its greater susceptibility to the neurotropism and neurovirulence of ZIKV infection.

Several recent studies provide some tractable experimental systems for modeling the impact of ZIKV on neurodevelopment and inspecting underlying cellular and molecular mechanisms.

The original ZIKV strain MR766 can directly infect NPCs derived from human induced pluripotent neural stem cells (hiPSCs) with high efficiency in vitro, and the infected hiNPCs also release infectious viral particles (Tang et al. 2016). ZIKV infection elicits dysregulation of cell cycle progression and cell loss of hiPSCs, attenuating their viability and growth as neurospheres and brain organoids (Garcez et al. 2016; Qian et al. 2016; Tang et al. 2016).

Animal models of human fetal infection are still needed to understand when, how, and why ZIKV invades developing brain, and provide the more detailed landscape of ZIKV-induced neurodevelopmental abnormalities. Cugola et al. (2016) inoculated Brazilian ZIKV strain into the brains of unborn SJL pups and detected cerebral malformations in the surviving fetuses, with decreased number of neural cells and cortical layer thickness, pathologies linked to fetal microcephaly in humans (Cugola et al. 2016). Upon close inspection of the ZIKV-infected mouse brains, Li et al. revealed that ZIKV^SZ01^ replicated in the embryonic mouse brain, suppressed proliferation and differentiation of NPCs after infection, ultimately leading to signs related with microcephaly (Li et al. 2016a). Wu et al. developed another mouse model by intraperitoneal injection of a contemporary ZIKV strain into the maternal mice, which provide evidence that ZIKV could be vertically transmitted from the pregnant mice to their fetuses (Wu et al. 2016). ZIKV invaded fetal brain results in a decrease in the proliferative pool of cerebral NPCs (Wu et al. 2016).

Neurotropic ZIKV could gain entry into the CNS and cause disease through many surface proteins, such as the candidate AXL receptor, which facilitates ZIKV to invade the developing CNS (Luethy et al. 2016; Nowakowski et al. 2016; Perera-Lecoin et al. 2013). Intriguingly, Nowakowski et al. (2016) assessed the expressive patterns of candidate ZIKV entry proteins by single-cell RNA-seq and revealed that conserved and high AXL expression in NPCs could render this neural cell population selectively susceptible to ZIKV infection (Nowakowski et al. 2016). Consistently, many candidate ZIKV entry receptors were significantly elicited, most strikingly AXL in animal models of ZIKV-induced microcephaly (Li et al. 2016a).

ZIKV infection elicits the deregulation of genes related to neurogenesis like Nestin and Sox2, differentiation, apoptotic pathways, and immune response-associated factors including TLR3, which has been validated in several independent in vivo and in vitro studies (Dang et al. 2016; Li et al. 2016a; Li et al. 2016b; Tang et al. 2016; Wu et al. 2016). Multiple key cellular signaling cascades, including the PI3K-Akt-mTOR pathway, are critical for neurogenesis from NPCs, as well as for subsequent migration and maturation, and autophagy regulation in brain development (Lee 2015; Takei and Nawa 2014). Liang et al. (2016) revealed that after invading of NPCs in the fetal brain, the nonstructural proteins NS4A and NS4B of ZIKV prohibit the Akt-mTOR cascade, disturbing neurogenesis and eliciting autophagy (Liang et al. 2016). Therefore, the above researches define candidate molecular targets of ZIKV pathogenesis and highlight potential determinants for pharmaceutical intervention.

## ZIKV linked pathologies beyond congenital microcephaly

ZIKV have numerous cellular tropisms and various factors that contribute to pathogenic consequences, involving defined cellular response and tissue accessibility (Diamond and Pierson 2015; Sips et al. 2012). ZIKV invades optic nerve, retina, iris, and cornea, and trigger panuveitis, conjunctivitis, and neuroretinitis in mice (Miner et al. 2016b). The results from human studies also reveal that ZIKV analogously infects the human ophthalmic tissues and trigger adverse eye illnesses including optic neuritis, chorioretinal atrophy, and blindness in neonates, and conjunctivitis and uveitis in adults (Miner et al. 2016b; Sun et al. 2016). Congenital ocular disease triggered by ZIKV could be due to direct invading of fetal ocular cells.

A notable rise in the incidence of GBS, observed in French Polynesia and Colombia during ZIKV outbreaks, respectively, in 2013 and 2016, implies ZIKV a pathogen that can lead to GBS, a severe neurological disease characterized by an immune-mediated progressive muscle weakness that can ultimately trigger flaccid paralysis and respiratory failure (Malkki 2016b; Parra et al. 2016; van den Berg et al. 2014). Moreover, ZIKV can produce neurological outcomes, including demyelinating polyneuropathy and brachial plexopathy and recently acute meningoencephalitis in adult (Carteaux et al. 2016). These neurological disorders may in fact be consequences of ZIKV exposure in adult.

Utilizing triply *Irf*-disrupted mice, Li et al. (2016b) revealed that blood-borne ZIKV administration can infect NPCs, causing cell death and decreased proliferation, in the adult neurogenic zones (Li et al. 2016b). We note that ZIKV also infects other human cell types, including skin cells and fibroblasts (Hamel et al. 2015). Intriguingly, an early mouse research observed ZIKV infection of neurons and astrocytes and detected enlarged astrocytes (Bell et al. 1971). ZIKV also invades immature neurons and astrocytes with lower efficiency in mice (Bell et al. 1971; Tang et al. 2016). These findings raise pivotal concerns about pathological effects on neurons and other cell types in the central and peripheral nervous system, as well as potential long-term outcomes.

## Conclusion

ZIKV-linked neuropathology is an eager global health alarm. Accumulated evidence from clinical, imaging, laboratory, and pathological assessment provided a more complete landscape of the severe neurological abnormalities triggered by ZIKV infection. After crossing the placental-fetal barrier, ZIKV breaches the fetal brain and invades neural stem cells as the primary target population with the highest AXL expression. By preferentially disrupting neural stem cells, the founder cells that generate all neuronal and glial cells, leading to neural cell death by autophagy and apoptosis, ZIKV can impair brain development and generate adverse microcephaly. The term congenital Zika syndrome is preferable to refer to these cases, as microcephaly is just one of the clinical signs of this congenital malformation abnormality. Moreover, results suggest that adult as well as fetal neural stem cells are susceptible to ZIKV infection. Broad cellular tropisms of ZIKV on numerous cell types could induce adverse long-term consequences.

## References

[CR1] Abbink P, Larocca RA, De La Barrera RA, Bricault CA, Moseley ET, Boyd M, Kirilova M, Li Z, Ng’ang’a D, Nanayakkara O (2016). Protective efficacy of multiple vaccine platforms against Zika virus challenge in rhesus monkeys. Science.

[CR2] Adams Waldorf, K.M., Stencel-Baerenwald, J.E., Kapur, R.P., Studholme, C., Boldenow, E., Vornhagen, J., Baldessari, A., Dighe, M.K., Thiel, J., Merillat, S.*, et al.* (2016). Fetal brain lesions after subcutaneous inoculation of Zika virus in a pregnant nonhuman primate. Nat Med 10.1038/nm.4193 PMC5365281 27618651

[CR3] Barba-Spaeth G, Dejnirattisai W, Rouvinski A, Vaney MC, Medits I, Sharma A, Simon-Loriere E, Sakuntabhai A, Cao-Lormeau VM, Haouz A (2016). Structural basis of potent Zika-dengue virus antibody cross-neutralization. Nature.

[CR4] Barkovich AJ, Guerrini R, Kuzniecky RI, Jackson GD, Dobyns WB (2012). A developmental and genetic classification for malformations of cortical development: update 2012. Brain.

[CR5] Baronti C, Piorkowski G, Charrel RN, Boubis L, Leparc-Goffart I, de Lamballerie X (2014) Complete coding sequence of zika virus from a French polynesia outbreak in 2013. Genome Announc 2 10.1128/genomeA.00500-14 PMC4047448 24903869

[CR6] Bell TM, Field EJ, Narang HK (1971). Zika virus infection of the central nervous system of mice. Arch Gesamte Virusforsch.

[CR7] Brasil, P., and Nielsen-Saines, K. (2016). More pieces to the microcephaly-Zika virus puzzle in Brazil. The Lancet Infectious diseases 10.1016/S1473-3099(16)30372-3 27641776

[CR8] Brasil P, Calvet GA, Siqueira AM, Wakimoto M, de Sequeira PC, Nobre A, Quintana Mde S, Mendonca MC, Lupi O, de Souza RV (2016). Zika virus outbreak in Rio de Janeiro, Brazil: clinical characterization, epidemiological and Virological aspects. PLoS Negl Trop Dis.

[CR9] Brasil, P., Pereira, J.P., Jr., Raja Gabaglia, C., Damasceno, L., Wakimoto, M., Ribeiro Nogueira, R.M., Carvalho de Sequeira, P., Machado Siqueira, A., Abreu de Carvalho, L.M., Cotrim da Cunha, D.*, et al.* (2016b). Zika virus infection in pregnant women in Rio de Janeiro—preliminary Report. The New England journal of medicine 10.1056/NEJMoa1602412 PMC5323261 26943629

[CR10] Broutet N, Krauer F, Riesen M, Khalakdina A, Almiron M, Aldighieri S, Espinal M, Low N, Dye C (2016). Zika virus as a cause of neurologic disorders. N Engl J Med.

[CR11] Budday S, Steinmann P, Kuhl E (2015). Physical biology of human brain development. Front Cell Neurosci.

[CR12] Calvet G, Aguiar RS, Melo AS, Sampaio SA, de Filippis I, Fabri A, Araujo ES, de Sequeira PC, de Mendonca MC, de Oliveira L (2016). Detection and sequencing of Zika virus from amniotic fluid of fetuses with microcephaly in Brazil: a case study. Lancet Infect Dis.

[CR13] Carteaux G, Maquart M, Bedet A, Contou D, Brugieres P, Fourati S, Cleret de Langavant L, de Broucker T, Brun-Buisson C, Leparc-Goffart I (2016). Zika virus associated with meningoencephalitis. N Engl J Med.

[CR14] Coyne CB, Lazear HM (2016). Zika virus—reigniting the TORCH. Nat Rev Microbiol.

[CR15] Cugola FR, Fernandes IR, Russo FB, Freitas BC, Dias JL, Guimaraes KP, Benazzato C, Almeida N, Pignatari GC, Romero S (2016). The Brazilian Zika virus strain causes birth defects in experimental models. Nature.

[CR16] Dang J, Tiwari SK, Lichinchi G, Qin Y, Patil VS, Eroshkin AM, Rana TM (2016). Zika virus depletes neural progenitors in human cerebral organoids through activation of the innate immune receptor TLR3. Cell Stem Cell.

[CR17] de Fatima Vasco Aragao M, van der Linden V, Brainer-Lima AM, Coeli RR, Rocha MA, Sobral da Silva P, Durce Costa Gomes de Carvalho M, van der Linden A, Cesario de Holanda A, Valenca MM (2016). Clinical features and neuroimaging (CT and MRI) findings in presumed Zika virus related congenital infection and microcephaly: retrospective case series study. BMJ.

[CR18] Diamond MS, Pierson TC (2015). Molecular insight into dengue virus pathogenesis and its implications for disease control. Cell.

[CR19] D’Ortenzio E, Matheron S, Yazdanpanah Y, de Lamballerie X, Hubert B, Piorkowski G, Maquart M, Descamps D, Damond F, Leparc-Goffart I (2016). Evidence of sexual transmission of Zika Virus. N Engl J Med.

[CR20] Dowd, K.A., Ko, S.Y., Morabito, K.M., Yang, E.S., Pelc, R.S., DeMaso, C.R., Castilho, L.R., Abbink, P., Boyd, M., Nityanandam, R.*, et al.* (2016). Rapid development of a DNA vaccine for Zika virus. Science 10.1126/science.aai9137 PMC5304212 27708058

[CR21] Faria NR, Azevedo Rdo S, Kraemer MU, Souza R, Cunha MS, Hill SC, Theze J, Bonsall MB, Bowden TA, Rissanen I (2016). Zika virus in the Americas: early epidemiological and genetic findings. Science.

[CR22] Garcez PP, Loiola EC, Madeiro da Costa R, Higa LM, Trindade P, Delvecchio R, Nascimento JM, Brindeiro R, Tanuri A, Rehen SK (2016). Zika virus impairs growth in human neurospheres and brain organoids. Science.

[CR23] Haddow AD, Schuh AJ, Yasuda CY, Kasper MR, Heang V, Huy R, Guzman H, Tesh RB, Weaver SC (2012). Genetic characterization of Zika virus strains: geographic expansion of the Asian lineage. PLoS Negl Trop Dis.

[CR24] Hamel R, Dejarnacphélie, Wichit S, Ekchariyawat P, Neyret A, Luplertlop N, Perera-Lecoin M, Surasombatpattana P, Talignani L, Thomas F, Cao-Lormeau V-M, Choumet V, Briant L, Desprès P, Amara A, Yssel H, Missé D, Diamond MS (2015) Biology of Zika virus infection in human skin cells. J Virol 89(17):8880–8896 10.1128/JVI.00354-15 PMC4524089 26085147

[CR25] Hayes EB (2009). Zika virus outside Africa. Emerg Infect Dis.

[CR26] Heymann DL, Hodgson A, Sall AA, Freedman DO, Staples JE, Althabe F, Baruah K, Mahmud G, Kandun N, Vasconcelos PF (2016). Zika virus and microcephaly: why is this situation a PHEIC?. Lancet.

[CR27] Hills SL, Russell K, Hennessey M, Williams C, Oster AM, Fischer M, Mead P (2016). Transmission of Zika virus through sexual contact with travelers to areas of ongoing transmission—continental United States, 2016. MMWR Morb Mortal Wkly Rep.

[CR28] Kriegstein A, Alvarez-Buylla A (2009). The glial nature of embryonic and adult neural stem cells. Annu Rev Neurosci.

[CR29] Kuno G, Chang GJ (2007). Full-length sequencing and genomic characterization of Bagaza, Kedougou, and Zika viruses. Arch Virol.

[CR30] Lanciotti RS, Kosoy OL, Laven JJ, Velez JO, Lambert AJ, Johnson AJ, Stanfield SM, Duffy MR (2008). Genetic and serologic properties of Zika virus associated with an epidemic, Yap State, Micronesia, 2007. Emerg Infect Dis.

[CR31] Lazear HM, Diamond MS (2016). Zika virus: new clinical syndromes and its emergence in the Western hemisphere. J Virol.

[CR32] Lazear HM, Govero J, Smith AM, Platt DJ, Fernandez E, Miner JJ, Diamond MS (2016). A mouse model of Zika virus pathogenesis. Cell Host Microbe.

[CR33] Lee DY (2015). Roles of mTOR signaling in brain development. Exp Neurobiol.

[CR34] Li C, Xu D, Ye Q, Hong S, Jiang Y, Liu X, Zhang N, Shi L, Qin CF, Xu Z (2016). Zika virus disrupts neural progenitor development and leads to microcephaly in mice. Cell Stem Cell.

[CR35] Li, H., Saucedo-Cuevas, L., Regla-Nava, J.A., Chai, G., Sheets, N., Tang, W., Terskikh, A.V., Shresta, S., and Gleeson, J.G. (2016b). Zika virus infects neural progenitors in the adult mouse brain and alters proliferation. Cell stem cell 10.1016/j.stem.2016.08.005 PMC5097023 27545505

[CR36] Liang, Q., Luo, Z., Zeng, J., Chen, W., Foo, S.S., Lee, S.A., Ge, J., Wang, S., Goldman, S.A., Zlokovic, B.V.*, et al.* (2016). Zika virus NS4A and NS4B proteins deregulate Akt-mTOR signaling in human fetal neural stem cells to inhibit neurogenesis and induce autophagy. Cell stem cell 10.1016/j.stem.2016.07.019 PMC5144538 27524440

[CR37] Luethy LN, Erickson AK, Jesudhasan PR, Ikizler M, Dermody TS, Pfeiffer JK (2016). Comparison of three neurotropic viruses reveals differences in viral dissemination to the central nervous system. Virology.

[CR38] Malkki H (2016). CNS infections: mouse studies confirm the link between Zika virus infection and microcephaly. Nat Rev Neurol.

[CR39] Malkki H (2016). CNS infections: Zika virus infection could trigger Guillain-Barre syndrome. Nat Rev Neurol.

[CR40] Mansuy JM, Pasquier C, Daudin M, Chapuy-Regaud S, Moinard N, Chevreau C, Izopet J, Mengelle C, Bujan L (2016). Zika virus in semen of a patient returning from a non-epidemic area. Lancet Infect Dis.

[CR41] Martines RB, Bhatnagar J, Keating MK, Silva-Flannery L, Muehlenbachs A, Gary J, Goldsmith C, Hale G, Ritter J, Rollin D (2016). Notes from the field: evidence of Zika virus infection in brain and placental tissues from two congenitally infected newborns and two fetal losses—Brazil, 2015. MMWR Morb Mortal Wkly Rep.

[CR42] Miner JJ, Cao B, Govero J, Smith AM, Fernandez E, Cabrera OH, Garber C, Noll M, Klein RS, Noguchi KK (2016). Zika virus infection during pregnancy in mice causes placental damage and fetal demise. Cel l.

[CR43] Miner, J.J., Sene, A., Richner, J.M., Smith, A.M., Santeford, A., Ban, N., Weger-Lucarelli, J., Manzella, F., Ruckert, C., Govero, J.*, et al.* (2016b). Zika virus infection in mice causes panuveitis with shedding of virus in tears. Cell Rep 10.1016/j.celrep.2016.08.079 PMC5040391 27612415

[CR44] Mlakar J, Korva M, Tul N, Popovic M, Poljsak-Prijatelj M, Mraz J, Kolenc M, Resman Rus K, Vesnaver Vipotnik T, Fabjan Vodusek V (2016). Zika virus associated with microcephaly. N Engl J Med.

[CR45] Molyneaux BJ, Arlotta P, Menezes JR, Macklis JD (2007). Neuronal subtype specification in the cerebral cortex. Nat Rev Neurosci.

[CR46] Mukhopadhyay S, Kuhn RJ, Rossmann MG (2005). A structural perspective of the flavivirus life cycle. Nat Rev Microbiol.

[CR47] Nowakowski TJ, Pollen AA, Di Lullo E, Sandoval-Espinosa C, Bershteyn M, Kriegstein AR (2016). Expression analysis highlights AXL as a candidate Zika virus entry receptor in neural stem cells. Cell Stem Cell.

[CR48] Parra, B., Lizarazo, J., Jimenez-Arango, J.A., Zea-Vera, A.F., Gonzalez-Manrique, G., Vargas, J., Angarita, J.A., Zuniga, G., Lopez-Gonzalez, R., Beltran, C.L.*, et al.* (2016). Guillain-Barre Syndrome associated with Zika virus infection in Colombia. The New England journal of medicine 10.1056/NEJMoa1605564 27705091

[CR49] Pastula DM, Smith DE, Beckham JD, Tyler KL (2016). Four emerging arboviral diseases in North America: Jamestown Canyon, Powassan, chikungunya, and Zika virus diseases. J Neurovirol.

[CR50] Perera-Lecoin M, Meertens L, Carnec X, Amara A (2013). Flavivirus entry receptors: an update. Viruses.

[CR51] Petersen E, Wilson ME, Touch S, McCloskey B, Mwaba P, Bates M, Dar O, Mattes F, Kidd M, Ippolito G (2016). Rapid spread of Zika virus in the Americas—implications for public health preparedness for mass gatherings at the 2016 Brazil Olympic Games. International journal of infectious diseases : IJID : official publication of the International Society for Infectious Diseases.

[CR52] Pierson, T.C., and Graham, B.S. (2016). Zika virus: immunity and vaccine development. Cell 10.1016/j.cell.2016.09.020 PMC5074878 27693357

[CR53] Prisant, N., Bujan, L., Benichou, H., Hayot, P.H., Pavili, L., Lurel, S., Herrmann, C., Janky, E., and Joguet, G. (2016). Zika virus in the female genital tract. The Lancet Infectious diseases 10.1016/S1473-3099(16)30193-1 27427201

[CR54] Qian X, Nguyen HN, Song MM, Hadiono C, Ogden SC, Hammack C, Yao B, Hamersky GR, Jacob F, Zhong C (2016). Brain-region-specific organoids using mini-bioreactors for modeling ZIKV exposure. Cell.

[CR55] Quicke KM, Bowen JR, Johnson EL, McDonald CE, Ma H, O’Neal JT, Rajakumar A, Wrammert J, Rimawi BH, Pulendran B (2016). Zika virus infects human placental macrophages. Cell Host Microbe.

[CR56] Rasmussen SA, Jamieson DJ, Honein MA, Petersen LR (2016). Zika virus and birth defects—reviewing the evidence for causality. N Engl J Med.

[CR57] Sarno, M., Sacramento, G.A., Khouri, R., do Rosario, M.S., Costa, F., Archanjo, G., Santos, L.A., Nery, N., Jr., Vasilakis, N., Ko, A.I.*, et al.* (2016). Zika virus infection and stillbirths: a case of hydrops fetalis, hydranencephaly and fetal demise. PLoS Negl Trop Dis 10, e0004517. 10.1371/journal.pntd.0004517 PMC4767410 26914330

[CR58] Sips GJ, Wilschut J, Smit JM (2012). Neuroinvasive flavivirus infections. Rev Med Virol.

[CR59] Sun, J., Wu, Zhong, H., Guan, D., Zhang, H., Tan, Q., and Ke, C. (2016). Presence of Zika virus in conjunctival fluid. JAMA Ophthalmol 10.1001/jamaophthalmol.2016.3417 27632055

[CR60] Suter MA, Aagaard KM (2016). Disease watch: Zika virus—placental passage and permissivity for infection. Nat Rev Endocrinol.

[CR61] Suthar MS, Diamond MS, Gale M (2013). West Nile virus infection and immunity. Nat Rev Microbiol.

[CR62] Tabata, T., Petitt, M., Puerta-Guardo, H., Michlmayr, D., Wang, C., Fang-Hoover, J., Harris, E., and Pereira, L. (2016). Zika virus targets different primary human placental cells, suggesting two routes for vertical transmission. Cell host & microbe 10.1016/j.chom.2016.07.002 PMC5257282 27443522

[CR63] Takei N, Nawa H (2014). mTOR signaling and its roles in normal and abnormal brain development. Front Mol Neurosci.

[CR64] Tang H, Hammack C, Ogden SC, Wen Z, Qian X, Li Y, Yao B, Shin J, Zhang F, Lee EM (2016). Zika virus infects human cortical neural progenitors and attenuates their growth. Cell Stem Cell.

[CR65] van den Berg B, Walgaard C, Drenthen J, Fokke C, Jacobs BC, van Doorn PA (2014). Guillain-Barre syndrome: pathogenesis, diagnosis, treatment and prognosis. Nat Rev Neurol.

[CR66] Wang L, Valderramos SG, Wu A, Ouyang S, Li C, Brasil P, Bonaldo M, Coates T, Nielsen-Saines K, Jiang T (2016). From mosquitos to humans: genetic evolution of Zika virus. Cell Host Microbe.

[CR67] Wikan N, Smith DR (2016). Zika virus: history of a newly emerging arbovirus. Lancet Infect Dis.

[CR68] Wilsch-Brauninger M, Florio M, Huttner WB (2016). Neocortex expansion in development and evolution—from cell biology to single genes. Curr Opin Neurobiol.

[CR69] Wollnik B (2010). A common mechanism for microcephaly. Nat Genet.

[CR70] Woodworth MB, Custo Greig L, Kriegstein AR, Macklis JD (2012). SnapShot: cortical development. Cell.

[CR71] Wu KY, Zuo GL, Li XF, Ye Q, Deng YQ, Huang XY, Cao WC, Qin CF, Luo ZG (2016). Vertical transmission of Zika virus targeting the radial glial cells affects cortex development of offspring mice. Cell Res.

